# Buffalo Medical Association

**Published:** 1858-11

**Authors:** 


### EDITORIAL DEPARTMENT.

---

*Buffalo Medical Association.*—By a glance of the proceedings of the Buffalo Medical Association which appear in this number, it will be seen that the resolutions which were presented to that body at the meeting before, on the subject of "*Criminal Abortions,*" have been unanimously adopted. This united action on the part of the society will, in our opinion, be productive of great good; and we feel flattered that the measures which we proposed for the abatement of this evil, in the September number of this Journal, were so unanimously approved. One of the resolutions which were adopted by the association, was that the coöperation of the Medical Societies in the State be invited, in any measures which we deemed expedient to take. As a member of the committee from the Buffalo Association we here invite the coöperation of other medical societies, and we beg that this matter may be brought before them as soon as possible. We shall send a copy of this number of the Journal, as well as one which contains our first editorial article, to members of every medical society which was represented at the last meeting of the State Society, in the hope and expectation that they will join with us either in the measures which we propose, or some which are better. We hope that every subscriber to this Journal, will not only bring the matter before the medical societies of which he is a member, but will interest himself to see it energetically acted upon. We should also be glad to receive communications relating to the matter with reports of cases where "abortion medicines" have been used. A few facts contributed in this way will do much towards awakening the mind of the public to the outrages practiced upon them.

